# Immunomodulation of cuproptosis and ferroptosis in liver cancer

**DOI:** 10.1186/s12935-023-03207-y

**Published:** 2024-01-10

**Authors:** Jia-qian Mo, Shen-yan Zhang, Qiang Li, Mo-xian Chen, Yue-qing Zheng, Xin Xie, Rongxin Zhang, Shan-shan Wang

**Affiliations:** 1 School of Life Sciences and Biopharmaceutics, Guang Dong Pharmaceutical University, Guangzhou, 51006 China; 2 State Key Laboratory of Tree Genetics and Breeding, Co-Innovation Center for Sustainable Forestry in Southern China and College of Life Sciences, Nanjing Forestry University, Nanjing, 210037 China; 3 Guang Zhou Zengcheng District Centre for Disease Control and Prevention, Guang Dong, 511300 China; 4 School of Life and Environmental Sciences, Shaoxing University, Shaoxing, 312000 Zhejiang China

**Keywords:** Cuproptosis, Copper, Ferroptosis, Liver cancer, Tumor microenvironment

## Abstract

According to statistics, the incidence of liver cancer is increasing yearly, and effective treatment of liver cancer is imminent. For early liver cancer, resection surgery is currently the most effective treatment. However, resection does not treat the disease in advanced patients, so finding a method with a better prognosis is necessary. In recent years, ferroptosis and cuproptosis have been gradually defined, and related studies have proved that they show excellent results in the therapy of liver cancer. Cuproptosis is a new form of cell death, and the use of cuproptosis combined with ferroptosis to inhibit the production of hepatocellular carcinoma cells has good development prospects and is worthy of in-depth discussion by researchers. In this review, we summarize the research progress on cuproptosis combined with ferroptosis in treating liver cancer, analyze the value of cuproptosis and ferroptosis in the immune of liver cancer, and propose potential pathways in oncotherapy with the combination of cuproptosis and ferroptosis, which can provide background knowledge for subsequent related research.

## Facts

1. Cuproptosis and ferroptosis, a form of programmed cell death, are closely related to cancer.
2. The immune microenvironment plays a crucial role in the occurrence and progression of liver cancer.
3. The tumor microenvironment (TME) has a complex structure and function and plays a key role in tumor development.

## Open questions

1. Is there a correlation between cuproptosis and ferroptosis?
2. Is there a correlation between immune and cuproptosis or ferroptosis?
3. What role does cuproptosis or ferroptosis play in liver cancer?

## Introduction

Liver cancer is the sixth most common malignancy worldwide and the second leading cause of premature death from cancer [1]. Liver cancer includes hepatocellular carcinoma (HCC), intrahepatic cholangiocarcinoma (CCA), and other rare types, of which HCC is the largest subtype of liver cancer [2]. In recent years, researchers have been working on various new ways to treat liver cancer. For instance, immunotherapies, such as cell-programmed death ligand-1 (PD-L1) inhibitor atezolizumab in combination with anti-angiogenic bevacizumab, have been shown to achieve good efficacy in the therapy of unresectable liver cancer [3]. IFN-α enhances the immune response against programmed death receptor-1 (PD-1) by remodeling the liver cancer glucose metabolism microenvironment [4]. Tsvetkov et al. discovered a new mode of cell death regulation and named it "cuproptosis". The emergence of cuproptosis has become a research hotspot in recent years, and its mechanism of action has been initially elucidated [5]. With the in-depth study of cuproptosis, it has shown excellent results in tumor therapy. At present, cuproptosis combined with ferroptosis has not been more comprehensively elaborated in the therapy of liver cancer, which provides a new idea for liver cancer therapy and can become a new therapeutic target and a reference for the development of drug candidates for the therapy of liver cancer. This article reviews the mechanism of cuproptosis, the correlation between cuproptosis and ferroptosis, the application of cuproptosis combined with ferroptosis in the therapy of liver cancer, and its prognostic value, and we discuss the development prospects and challenges of cuproptosis combined with ferroptosis.

## Mechanism of cuproptosis and ferroptosis

With the in-depth study of the mechanism of mediating programmed cell death (PCD), many new forms of PCD have been discovered, including apoptosis, necrotizing apoptosis, pyroptosis, high-profile ferroptosis, and, more recently, cuproptosis. Unwanted cells are cleared by PCD through gene regulation to maintain homeostasis in multicellular organisms. Through PCD, we have a deeper understanding of the mechanisms by which cancer occurs [6]. For example, ferroptosis is a form of cell death driven by iron-dependent lipid peroxidation, discovered in Brent Stockwell’s lab in 2012 [7]. Blocking the process of converting phospholipid hydroperoxide (PLOOH) by the ferroptosis regulator GPX4 leads to the accumulation of PLOOH and produces a tumor suppressor effect [8]. In the early days, the existence of cuproptosis was implicated in experimental studies. Cuproptosis is a programmed death of cells due to the inhibition of mitochondrial respiratory regulation. Fe-S cluster proteins are electron carriers during mitochondrial respiration, and they play the role of transmitting electrons. Copper can reduce the formation of Fe-S cluster proteins by inhibiting the synthesis of mitochondrial-associated proteins [9]. Mechanistically, the copper direct binding of thioylated proteins leads to their oligomerization, followed by a loss of Fe-S cluster proteins, which affects the regulation of mitochondrial respiration and eventually induce a proteotoxic stress response, causing cell death [5, 10]. The mechanism of cuproptosis provides a strong frontier of technical support for the study of new methods for the therapy of cancer, and it is expected to be combined with ferroptosis to become a new way to treat cancer in the future.

## Copper transporter and iron transporter in liver cancer

### Copper transporter in liver cancer

Copper is an important trace element in the human body, playing roles in energy conversion, growth metabolism, cellular respiration, redox, and more. The dysregulation of copper metabolic homeostasis can cause damage to cells and affect tumor growth. It is known that the serum of cancer patients has higher levels of copper than that of healthy people, indicating that copper affects human health [11–14]. Further research is still ongoing on the metabolism of copper in hepatocytes and its cancer-promoting effects. Copper uptake outside the cell is performed by copper transporter 1 (CTR1, encoded by SLC31A1), and the expression of CTR1 prevents excessive copper from entering the cell. Studies have proved that CTR1 is highly expressed in the liver tissues of liver cancer patients, and CTR1 mediates copper to indirectly affect the cell signaling cascade and promote tumorigenesis and development [15, 16]. Jianping, G. et al. revealed that copper ions activate protein kinase B (PKB, also known as AKT) through CTR1 and demonstrated that copper can bind phosphoinositol-dependent protein kinase 1 (PDK1) and enhance the interaction between PDK1 and AKT, thereby activating phosphoinositol kinase 3 (PI3K)-AKT Carcinogenic signaling pathways promote the occurrence of cancer [17]. SLC31A1 is equivalent to the input of copper. The inhibition of SLC31A1-dependent copper transport leads to anti-cell-death effects in cancer cells [18]. Studies have found that the expression of SLC31A1 in liver cancer samples has changed, which is related to the increase in copper content in liver tissues; liver cancer cell lines rely on CTR1 to transport copper for proliferation, colony formation, and growth. Additionally, studies have revealed that under hypoxic conditions, the loss of CTR1 intensifies and reduces liver cancer cell viability [19].

The ATP7A and ATP7B genes provide important instructions for regulating copper levels in vivo, and they have the function of exporting copper to maintain copper homeostasis within cells. The superoxide dismutase copper chaperone (CCS) transports copper to superoxide dismutase-1 (SOD1), and the copper ion transporter ATOX1 transports copper to ATP7A and ATP7B. When the copper content in the cellular environment is too high, ATP7A and ATP7B move to the cell membrane to eliminate excess copper [14]. The copper-dependent lysyl oxidase (LOX) family contributes to tumor metastasis, and its activity is dependent on the expression of ATP7A. Shanbhag, V. et al. demonstrated that blocking ATP7A copper transport can inhibit LOX activity, inhibit tumor growth and metastasis, and manifest as copper transport disruption when ATP7A is missing [20]. Aubert, L. et al. found that copper content was significantly elevated in KRAS mutant cells; this was due to the high expression of ATP7A in cancer cells, which reduced the sensitivity of cells to increase the copper; ATP7A is mostly present on the cell surface, maintains stable intracellular copper levels, and protects cancer cells from copper ion toxicity, thereby reducing the occurrence of cuproptosis [21]. Shao Ke et al. concluded that ATP7A can provide sufficient copper ions to oncogenic enzymes, mediate tumor immune escape, and promote tumor cell invasion by affecting immune cell infiltration and immune checkpoint expression in the tumor microenvironment, so ATP7A has unique prognostic significance [22].

Studies have shown an association between the copper transporter ATP7A and iron. Elesclomol (ES) induces ferroptosis in colorectal cancer (CRC) by degrading ATP7A; ES induces copper chelation, then increases copper levels while reducing ATP7A expression, which leads to reactive oxygen species (ROS) accumulation and promotes the degradation of SLC7A11. As a result, it enhances the oxidative stress of cells, leading to ferroptosis, and inhibits the antioxidant capacity of CRC cells [23]. Sorafenib is currently the only inducer that promotes ferroptosis in patients with liver cancer [24]. In past studies, it was revealed that it inhibits glutathione (GSH) production by inhibiting the cystine/glutamate reverse transport system (system x_c_^−^) to promote ferroptosis [25]. By analyzing liver cancer patients receiving sorafenib, some researchers found that patients with high ATP7A expression had a longer overall survival (OS), indicating that ATP7A can improve the therapeutic effect of sorafenib in liver cancer; that is, high ATP7A indirectly promotes ferroptosis in liver cancer [22].

In the liver, ATP7A and ATP7B have slightly different mechanisms of action. ATP7A helps HepG2 cells detoxify copper, allowing copper to be rapidly excreted from liver cells; however, ATP7B has a slower copper output rate, which reflects the mode of action of ATP7A and ATP7B in maintaining copper homeostasis in liver cells. Experiments on liver cancer have demonstrated elevated expression of ATP7A in liver cancer samples [26]. The increase in copper content in liver cancer cell tissue is due to the loss of ATP7B, rather than SLC31A1 and SLC31A2 down-regulation; and in the liver, the lack of ATP7B affects its cell cycle and lipid metabolism [19, 22, 27].

### Iron transporter in liver cancer

Iron is one of the critical metals for several vital biological processes. Persistent iron deficiency has been associated with fatigue, poorer cognitive and motor skills, defective immune cell function, and increased disease severity in heart failure [28]. Furthermore, iron deficiency promotes liver cancer metastasis [29]. The iron-transporting membrane protein ferroportin 1 (FPN1), the only known cellular exporter of iron, plays major roles in maintaining iron homeostasis. Several studies have shown that FPN1 is less abundant in cancer cells than in non-cancer cells, suggesting that FPN1 levels might play a role in cancer development [30]. A previous study showed that the knockdown of two iron metabolism genes (FPN and LCN2) in HepG2 cells induced ferroptosis and inhibited cell viability [31]. The transferrin receptor (TFR) is involved in the transport of iron into the hepatocytes, while iron transport out of the hepatocytes is regulated by ferroportin. Adachi et al. showed that a higher expression of TFR1 in liver cancer tissue was significantly associated with poor prognosis in liver cancer patients [32]. Furthermore, Krüpple-Like factor 14 (KLF14) can cause TFR1 downregulation and ferritin upregulation, which leads to cellular iron deficiency and hepatocellular carcinoma cell growth suppression in vitro and in vivo [33].

## Association between cuproptosis and ferroptosis

### Correlation between copper and GSH, an important antioxidant substance for ferroptosis

Recently, the mechanism between cuproptosis and ferroptosis was clearly explained, the relationship between the two is gradually becoming clear, and various analyses have shown a strong link between cuproptosis and ferroptosis regulators [34].

GSH is an important antioxidant in ferroptosis [35]. It is also one of the copper companions. Additionally, its downward regulation promotes the occurrence of cuproptosis [5]. NADPH is used to maintain the reducing property of glutathione and is downregulated in ferroptosis [35, 36], which indicates that GSH exists in the form of oxidation in ferroptosis. Redox homeostasis and lipid peroxidation are known to be important occurrence conditions in ferroptosis. The depletion of GSH and decreased activity of glutathione peroxidase GPX4 can lead to lipid peroxidation and metabolic dysfunction, increasing the sensitivity of tumor cells to ferroptosis [24]. ROS can support the rapid development of cancer cells, while GSH is used to remove excess ROS to maintain the balance of the redox state within the cell. But high levels of ROS trigger cell death. Therefore, in cancer cells, the loss of GSH makes the tumor more susceptible to oxidative stress, which plays an important role in improving ferroptosis. Furthermore, a strategy called GSH depletion has been proposed to illustrate its benefits in cancer therapy [37]. For instance, Zhanwei, Z. et al. prepared vesicles based on cinnamaldehyde dimer (CDC dimer), which can consume GSH in mouse breast cancer cells, while breaking itself and promoting vesicle release of drugs, enhancing the ferroptosis and immunotherapy effect of cancer cells [38]. Copper may promote ferroptosis through oxidative stress and the combination of ES and copper increases lipid ROS levels, which can consume large amounts of GSH and induce intense oxidative stress [23].

Free copper ions in cells may produce ROS. To avoid cytotoxicity, GSH binds to excess copper ions to ease intracellular copper levels, which determines the distribution of copper within the cell. On the other hand, GSH affects the copper output of ATP7A and ATP7B by maintaining the redox reaction of ATP7A and ATP7B cysteine [39]. To verify cuproptosis, researchers treated cells using butathionate sulfoxide (BSO) in combination with ES. BSO has the effect of consuming GSH, but GSH cannot chelate copper ions; that is, Cu2^+^-ES complexes are formed, which are transported to mitochondria for redox reactions, thereby forming oxidative stress and resulting in cuproptosis [5]. Similarly, regarding copper and oxidative stress, Huan, L. et al. demonstrated that excessive copper intake by hepatocytes can induce oxidative stress, increase ROS levels, and reduce the content of GSH in the liver by studying the effects of copper on oxidative stress and its effects in mouse livers [40]. Weiping, X.’s team developed a synergistic strategy based on cuproptosis, ferroptosis, and apoptosis to achieve anti-tumor effects. They established a copper-chelated dithiocarbamate and artemisinin-loaded hollow nano platform (HNP). Disulfide-rich HNP triples the oxidative stress produced by enlarged cells by consuming GSH in cells and enhancing the sensitivity of cancer cells to cuproptosis, while HNP inhibits the activity of GPX4, thereby activating ferroptosis [41].

The above shows that there is a strong correlation between copper and GSH, an important substance of ferroptosis. There are broad research prospects for the role of oxidative stress in the combination of cuproptosis and ferroptosis in the therapy of tumors. More studies have found that intracellular copper has a direct effect on GPX4, which plays an important role in ferroptosis as a regulator of ferroptosis, and excess copper binds to specific proteins on the surface of GPX4 to induce GPX4 oligomerization, which is a new way for copper to promote ferroptosis [42].

### The role of mitochondria in cuproptosis and ferroptosis

**Fig. 1:**
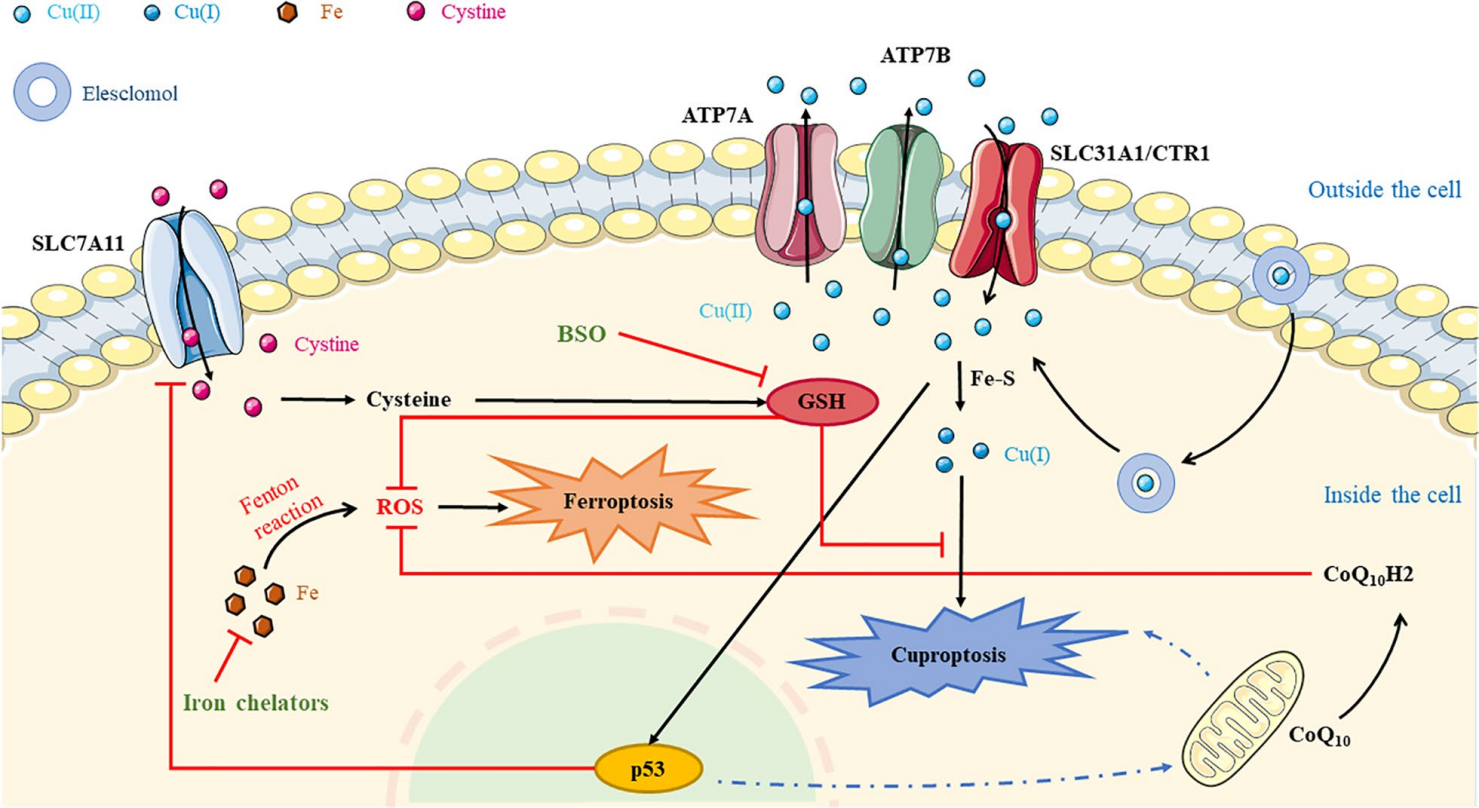
Association between Cuproptosis and Ferroptosis. Glutathione (GSH) inhibits cuproptosis; on the other hand, it can inhibit ferroptosis by inhibiting ROS. High levels of copper induce activation of the tumor suppressor gene p53, the activity of which may promote cuproptosis by enhancing mitochondrial function; p53 can inhibit the expression of the SLC7A11 gene, affect the balance of ROS and GSH, reduce cell uptake of cystine, and reduce cell antioxidant capacity, thereby promoting ferroptosis in tumor cells. Moreover, coenzyme Q10 (CoQ10) on the mitochondria is reduced to reduced coenzyme Q10 (CoQ10H2), which may inhibit ROS and lead to ferroptosis inhibition

Mitochondria are essential for cuproptosis and ferroptosis. Cuproptosis is known to be a proteotoxic stress due to blockage of the TCA cycle, while ferroptosis is also affected by the TCA cycle. Mitochondrial respiration is affected by the interaction between the cellular antioxidant system and glutamine catabolism regulating the TCA cycle. Uncontrolled production of ROS by mitochondrial respiration is key to cysteine deprivation (CDI)-induced ferroptosis. The depletion of GSH leads to the weakening of the function of cells to eliminate ROS, and then cell redox homeostasis disorders and ferroptosis happen, indicating that the inhibition of TCA cycling in mitochondria weakens the sensitivity of cells to ferroptosis [43] (Fig. 1). Moreover, coenzyme Q10 (CoQ10) on the mitochondria is reduced to reduced coenzyme Q10 (CoQ10H2), which may inhibit ROS and lead to ferroptosis inhibition. On the other hand, ferroptosis activators inhibit CoQ10 production [44].

### Copper homeostasis and iron homeostasis in the liver

Maintaining normal levels of copper ions in the body depends on different types of protein interactions. Ceruloplasmin (CP) is the main protein carrier used in plasma for the exchange of copper ions [45, 46], which is not only involved in ferroptosis but also in cuproptosis. Unlike maintaining copper homeostasis in the liver, CP controls iron homeostasis in the liver and thus inhibits liver cancer ferroptosis [24]. In hepatic iron homeostasis, the ceruloplasmin-ferroportin system (CP-FPN) is the main iron export system of hepatocytes, and the lack of CP promotes iron-mediated ROS production and erastin-induced ferroptosis [47]. Specific proteins are used to assist tumor cells in the uptake and excretion of copper ions and mediate the distribution of copper inside and to outside the cell. The copper transporters CTR1 and CTR2, the initiators of copper ions, are responsible for the process of uptaking copper and transporting it across the membrane. ATP7A and ATP7B, the exporters of copper ions, are responsible for expelling copper bound to the copper-binding site from the cell and avoiding copper overload [46]. Copper chaperones are divided into cytoplasmic copper chaperones (ATOX1, CCS) and mitochondrial copper chaperones (COX11, COX17, SCO1, SCO2), which are used to bind copper and mediate its insertion into ATP7A and ATP7B [48]. Copper-binding proteins MT1 and MT2 have a high affinity for copper ions and can chelate copper to prevent cell damage [48]. These proteins maintain the stability of copper ion content in the human body, and the dysregulation of copper homeostasis can lead to disturbances in cell metabolism, as well as cell death [45, 46, 48].

It has been shown that tumor suppressors are associated with copper homeostasis and play an important regulatory role in cuproptosis, mainly by inhibiting glycolysis and increasing the sensitivity of cells to cuproptosis [5, 49]. High levels of copper induce tumor suppressor gene P53 activation, and the activity of P53 may promote cuproptosis by enhancing mitochondrial function, so the mutation or deletion of P53 reduces COX activity as well as mitochondrial respiration [50]. While P53 is also confirmed to be involved in the synthesis of Fe-S cluster proteins, FDXR, ISCU, and FXN genes can encode related proteins for combining Fe-S cluster proteins. P53 can regulate the expression of the above three genes, which indicates that P53 is related to copper homeostasis and can be used to induce cuproptosis [50].

In ferroptosis, P53 also promotes the emergence of a new pattern of ferroptosis, and it has been shown that P53 can damage GSH in the event of oxidative stress by inhibiting the expression of the SLC7A11 gene [8], reduce the cell uptake of cystine, reduce the cellular antioxidant capacity, thereby promoting ferroptosis in tumor cells, and inhibit ferroptosis by negatively regulating dipeptidyl peptidase-4 (DDP-4) [36].

Copper homeostasis disorders in the human body can lead to human disease. For example, Wilson’s Disease (WD) is a classic example of excess copper levels, manifested in the liver by the inactivation of the copper transporter ATP7B and the continuous accumulation of copper, resulting in DNA damage, lipid peroxidation, and mitochondrial dysfunction. This can lead to acute liver failure, acute or chronic hepatitis, and cirrhosis [45, 51]. In patients with cirrhosis or chronic hepatitis, the long-term instability of copper levels leads to the development of liver cancer [52].

The imbalance of copper homeostasis and iron homeostasis in hepatocellular carcinoma cells can affect tumor development and drug resistance. The Warburg effect in liver cancer patients inhibits mitochondrial respiration. Furthermore, tumors are more inclined to obtain the required energy in the form of glycolysis, and the downregulation of TCA in combination with genetic mutations leads to the spread of liver cancer by reducing cell differentiation [53].

## Immunomodulation of cuproptosis and ferroptosis in liver cancer

### Mechanisms by which copper induces tumorigenesis associated with the immune system

Copper is one of the mineral elements that affects the body’s immune response and is associated with the immune system [54].

Before the term "cuproptosis", studies had shown that there was a significant increase in the copper ion concentration in different types of tumors, and reducing copper content could inhibit the formation of new blood vessels, thereby inhibiting tumorigenesis [48, 55]. Copper is involved in the mechanism of angiogenesis [56] and the relationship between copper and neovascularization may reveal why copper accumulates in the environment surrounding the tumor. Some researchers have developed a tumor-targeted micelle system based on amphiphilic polypeptides, which can deliver copper chelators and chemotherapy drugs to tumors at the same time. The content of copper in tumors is significantly reduced after treatment, reducing the neovascularization that supports tumor growth to regulate the tumor microenvironment [57]. Liver cancer is a highly vascularized cancer in which vascular endothelial growth factor (VEGF) suppresses the immune response by modulating immune cells (cytotoxic T cells, dendritic cells, Tregs, and MDSCs). Therefore, a combination of neurogenesis blockade and immunotherapy may be a promising therapy strategy [58].

Copper can also help tumors spread by activating cancer-cell-associated metastasis signals [51]. Studies have shown that copper chaperone ATOX1 is involved in the ATP7A-LOX pathway associated with tumor metastasis, and the inhibition of ATOX1 expression reduces LOX activity and the rate of cancer cell metastasis [59]. The immune checkpoint inhibitor PD-L1 is involved in the immune evasion of tumors, consuming copper to degrade PD-L1, which can inhibit tumor growth [60]. Recent studies have shown that high expression of ferredoxin 1 (FDX1), a cuproptosis-related genes, was associated with displayed higher immune cell infiltration and lower PD-L1, and decreased cell viability in liver cancer samples [61] (Table 1).

**Table 1:** Immunomodulation of cuproptosis and ferroptosis in liver cancer

Classification	Factor	Results	Type	References
Cuproptosis	FDX1	High FDX1 expression displayed higher immune cell infiltration and lower PD-L1	In vivo	[61]
	DLAT	Increasing the immune infiltration of Tregs, then, increases tumorigenesis and tumor invasion	In vivo	[62]
Ferroptosis	SLC7A11	Bound by CD8 + , then, inhibits tumor growth	In vivo	[63, 64]
	APOC1	Low APOC1 turns M2 into M1, then, inhibits tumor growth	In vivo	[65]
	GPX4	Tregs lack of GPX4 increases anti-tumor immunity	In vivo	[66, 67]

### Ferroptosis in immunomodulation

Since the advent of ferroptosis, a growing number of studies have found an association between ferroptosis and immune regulation. For example, some studies have shown that low expression of IFNγ manifests as a high metastasis rate and high recurrence rate of liver cancer, and activating CD8 + T cells to release IFNγ reduces the expression level of SLC7A11 and inhibits the cellular uptake of cystine, causing ferroptosis [63, 64]. A deficiency of APOC1 can inhibit liver cancer by inversely transforming M2 macrophages (TAM) into M1 macrophages through the ferroptosis pathway, increasing tumor sensitivity against PD-1 development [65]. The deficiency of GPX4 in regulatory T cells (Tregs) leads to the occurrence of ferroptosis and promotes the production of IL-1β, thereby increasing anti-tumor immunity [66]. Furthermore, Peter Vandenabeele et al. revealed that after inducing ferroptosis, tumor cells that develop ferroptosis have an inhibitory effect on the surrounding antigen-presenting cells [67]. Wentao Zeng et al. showed that ROS generation by NADPH oxidases (NOXs) is essential for the phagocytic and tumoricidal effects of M1 macrophages. Fatty acid oxidation (FAO) blockade reversed the immunosuppressive activity of tumor-associated macrophages (TAMs) and dampened liver cancer tumorigenesis. However, spermidine therapy enhanced the production of mitochondrial ROS to activate AMPK, which led to the upregulation of the components in FAO, as well as the total mass of mitochondria [68].

### Immune microenvironment in liver cancer

The immune microenvironment plays a crucial role in the occurrence and progression of liver cancer. The body’s immune system plays a vital role in the therapy of liver cancer. As an important part of innate immunity, the liver affects the occurrence of various liver diseases in its immune microenvironment. In an inflamed liver, a sustained immune response leads to hepatic immune microenvironment immunological failure, resulting in immunosuppression. The presence of an immunosuppressive liver microenvironment may be responsible for liver cancer’s ability to perform immune evasion [69].

The tumor microenvironment (TME) has a complex structure and function and plays a key role in tumor development. Cuproptosis and ferroptosis are closely related to the tumor immune microenvironment, and the TME contains a variety of cell types, including cancer cells, immune cells, endothelial cells, inflammatory cells, and fibroblasts (CAFs). In the TME, tumor cells interact with immune cells to regulate the process of cancer development. CAF is abundant in TME. In the process of liver cancer, along with the aggregation of CAF, CAF interacts with TME immune infiltrating cells through effector molecules, which can transform TME into an immunosuppressive type, help tumor cells evade immune surveillance, and carry out immune escape, indicating CAF provides an immune microenvironment for liver cancer that is conducive to its proliferation and metastasis [11, 70, 71]. Several long noncoding RNAs (lncRNAs) have been found to shape the tumor microenvironment through epigenetic regulation in tumor cells [72]. The production of ROS is associated with the release of malignant tumors [51]. ROS produced by copper excess promotes the release of damage-associated molecules (DAMPs), leading to immune dysfunction [11]. However, in ferroptosis, iron produces lipid ROS through the Fenton reaction, causing lipid peroxide aggregation and inducing ferroptosis in tumor cells [24].

As a prognostic factor for ferroptosis, the overexpression of SLC7A11 can hinder ferroptosis. Patients with liver cancer showed high levels of immune cells associated with poor prognosis with SLC7A11 [73]. Ferroptosis combined with ICIs is a new strategy for the therapy of liver cancer. Regulating the ferroptosis of different immune cells in the liver microenvironment and improving the expression of PD-L1 and T cell infiltration can increase anti-tumor immunity [8]. Additionally, a study has shown a correlation between cuproptosis-related dihydrolipoamide S-acetyltransferase (DLAT) and CD49d, which is one of the typical phenotype markers of Tregs. The high expression of DLAT may increase the immune infiltration of Tregs; thus, tumorigenesis and tumor invasion are ultimately promoted [62]. In sum, both cuproptosis and ferroptosis are involved in the regulation of TME in liver cancer; but for now, few currently verify the effect of cuproptosis federation with ferroptosis on TME in liver cancer.

## Value of cuproptosis combined with ferroptosis in predicting immune response and prognosis

It is necessary for therapy to predict the effects of cuproptosis and ferroptosis. Changwu Wu et al. found through data analysis that, although high cuproptosis activity reduced the immune response, it still improved the prognosis of a variety of tumors. Hhigh immunity does not mean a good prognosis, which is related to the characteristics of the tumor. The prognosis of high cuproptosis may be related to low stromal infiltration and low immunosuppression [74]. Liver cancer has wide heterogeneity and and therapy options currently available for liver cancer are limited. To accurately determine the therapeutic effect of cuproptosis on liver cancer, some researchers have established a related prognostic model for the therapeutic effect of cuproptosis in liver cancer through bioinformatics analysis and obtained a cuproptosis-related risk score (CRRS) [75–77]. These models all classify liver cancer patients into high-risk and low-risk groups. Analyses have shown that the expression level of cuproptosis-related genes (CRGs) in liver cancer can be regulated by methylation, and CRGs can lead to the development of liver cancer by affecting the tumor microenvironment [75]. FDX1 is a key regulator of cuproptosis, and the severity of liver cancer gradually increases, indicating that patients with a low expression of FDX1 have poorer OS than patients with a high expression of FDX1 [76]. The metabolic pathway of cuproptosis in the low-risk group was mainly clustered in TCA. Liver cancer in the high-risk group showed insensitivity to cuproptosis, and the TCA pathway was inhibited, indicating that the gene expression required for hypoxia or glycolysis in the high-risk group was higher than that in the low-risk group [76]. The CRRS is positively correlated with activated memory T cells and negatively with NK cells as well as Tregs. The presence of NK cells is associated with good prognosis; the presence of more NK cells is associated with longer OS, and fewer NK cells with shorter [75]. Patients in the high-risk group were characterized by elevated Treg expression associated with cancer-associated fibroblast (CAF) activation and higher tumor immune cell infiltration [76]. A high expression of immune checkpoint inhibitors (ICIs) is also characteristic of the high-risk group. Because of the high expressions of PD-L1, PD-1, cytotoxic T lymphocyte-associated protein-4 (CTLA-4), and so on, the high-risk group, exhibited in ICIs, compared to the low-risk group is more likely to benefit [76]. FNBP4 is associated with the TCA process of cuproptosis. After data analysis, it was found that the expression of FNBP4 in liver cancer increased significantly, and a high expression of FNBP4 was negatively correlated with the patient’s OS [78]. A cuproptosis-related gene (CRG) model was constructed by Ke, C. et al. Through clinical sample qPCR and immunohistochemical detection, it was determined that DLAT had a cancer-promoting effect, which was significantly upregulated in liver cancer and promoted HepG2 and Huh7 cell proliferation, migration, and invasion [79].

In ferroptosis, relevant prognostic models have also been established to predict the prognosis of patients. Studies have found that the ferroptosis genes ABCB6, FLVCR1, SLC48A1, and SLC7A11 have excellent predictions through analysis, and M0 macrophages, follicular helper T cells (Tfh), memory B cells, and neutrophils account for a relatively large proportion of high-risk-score liver cancer patients [80]. A composite index of ferroptosis and immune status (CIFI) was constructed by the researchers. Patients with high CIFI values showed that high-expression ferroptosis inhibitors and immunosuppressive cells, such as SLC7A11, significantly upregulated and significantly increased proportion of CAFs. Furthermore, M0 macrophages, plasma cells, neutrophils, and regulatory T cells (Tregs) were significantly increased, indicating that patients with high CIFI values have a worse prognosis in liver cancer [81]. By constructing a characteristic model of ferroptosis-related lncRNAs, it was found that the expression level of ICIs is higher in liver cancer patients in the high-risk group, indicating that ferroptosis-related lncRNAs can predict the expression level of ICIs. To improve the prognosis of high-risk patients, ICI and ferroptosis inducers can be combined to promote tumor cell ferroptosis [82]. A prognostic model with PRDX1 as the central gene was established, and it was found that it was highly expressed in liver cancer; especially in HepG2 cells, as an important antioxidant protein. PRDX1 was downregulated to verify its role in ferroptosis, and it was found that downregulating PRDX1 can promote ferroptosis, illustrating that PRDX1 can be used as a prognostic marker for liver cancer [83].

The above results were only predicted by computer data analysis, and their authenticity needs to be verified in the real world to determine their clinical effects. Nevertheless, establishing a prediction model based on the combination of cuproptosis and ferroptosis will provide data support for the therapy of liver cancer patients and a reference for clinical trials. For instance, combining cuproptosis with ferroptosis may efficiently regulate immune-related molecules and cells, and thus inhibit tumor development. At the same time, we should continue to establish more powerful and accurate prediction models to reveal the role of cuproptosis combined with ferroptosis on the development of liver cancer and find more effective therapies.

## Discussion

The discovery of cuproptosis is both an opportunity and a challenge. The new cell death mechanism provides a new therapeutic target for liver cancer. Importantly, the mechanism of cuproptosis and ferroptosis shows their great potential in tumor therapy. The rational use of the mechanism of cuproptosis combined with ferroptosis, for instance, by designing drugs that can inhibit the spread of liver cancer cells without systemic toxicity according to the sensitivity of tumors to cuproptosis and ferroptosis, may establish a good prognosis for cancer patients.

**Fig. 2:**
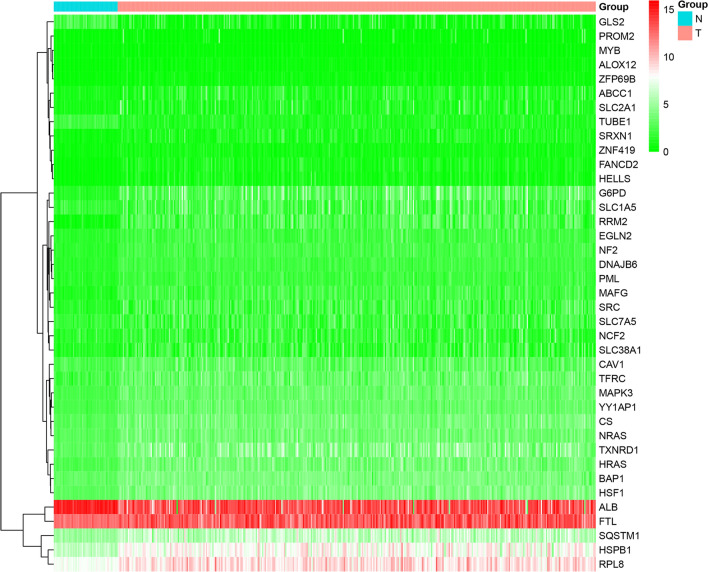
Heatmap of the expression level of cuproptosis-related ferroptosis genes in liver cancer datasets

**Fig. 3:**
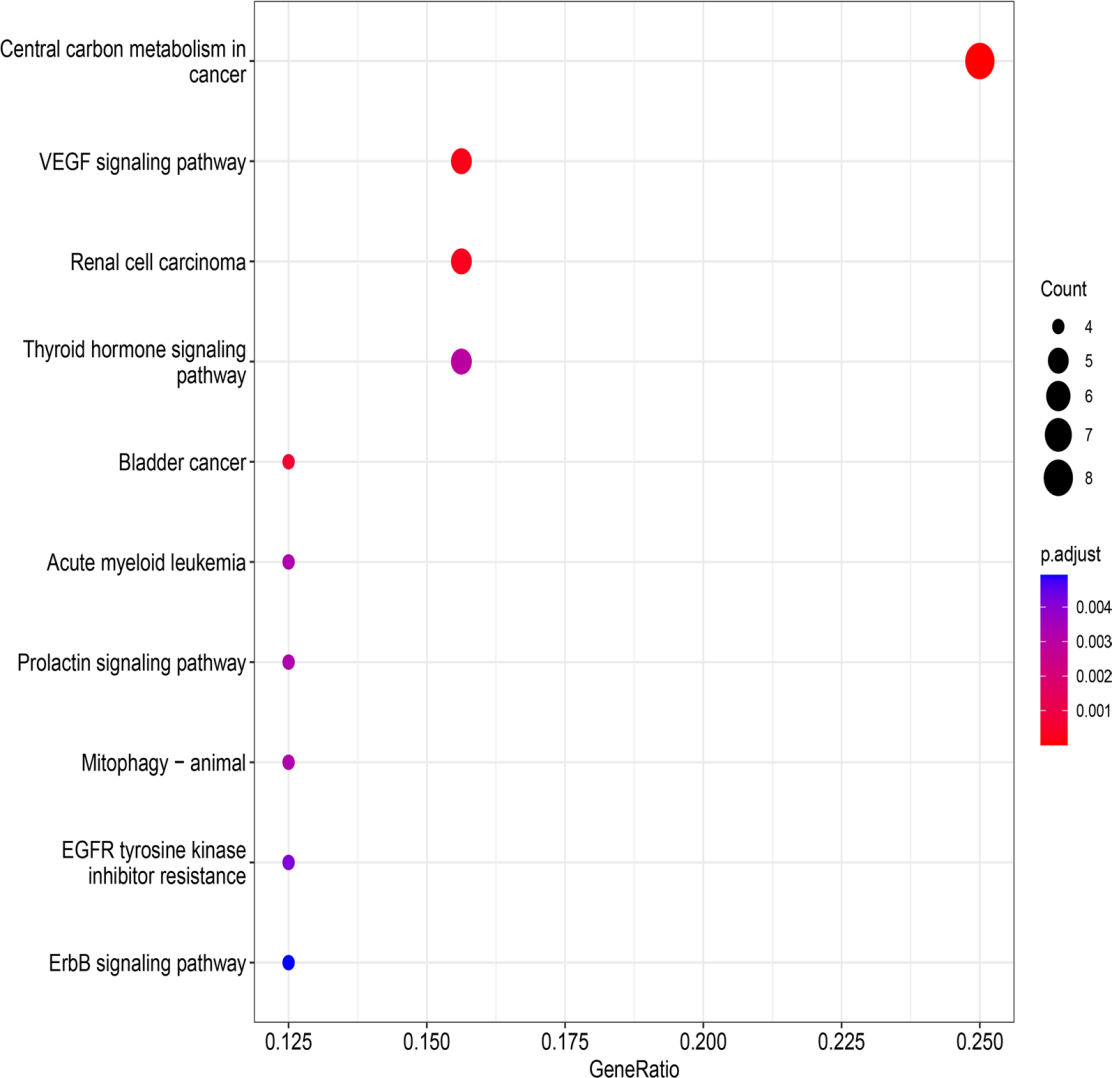
Results of cuproptosis-related ferroptosis genes from KEGG pathway analysis

**Fig. 4:**
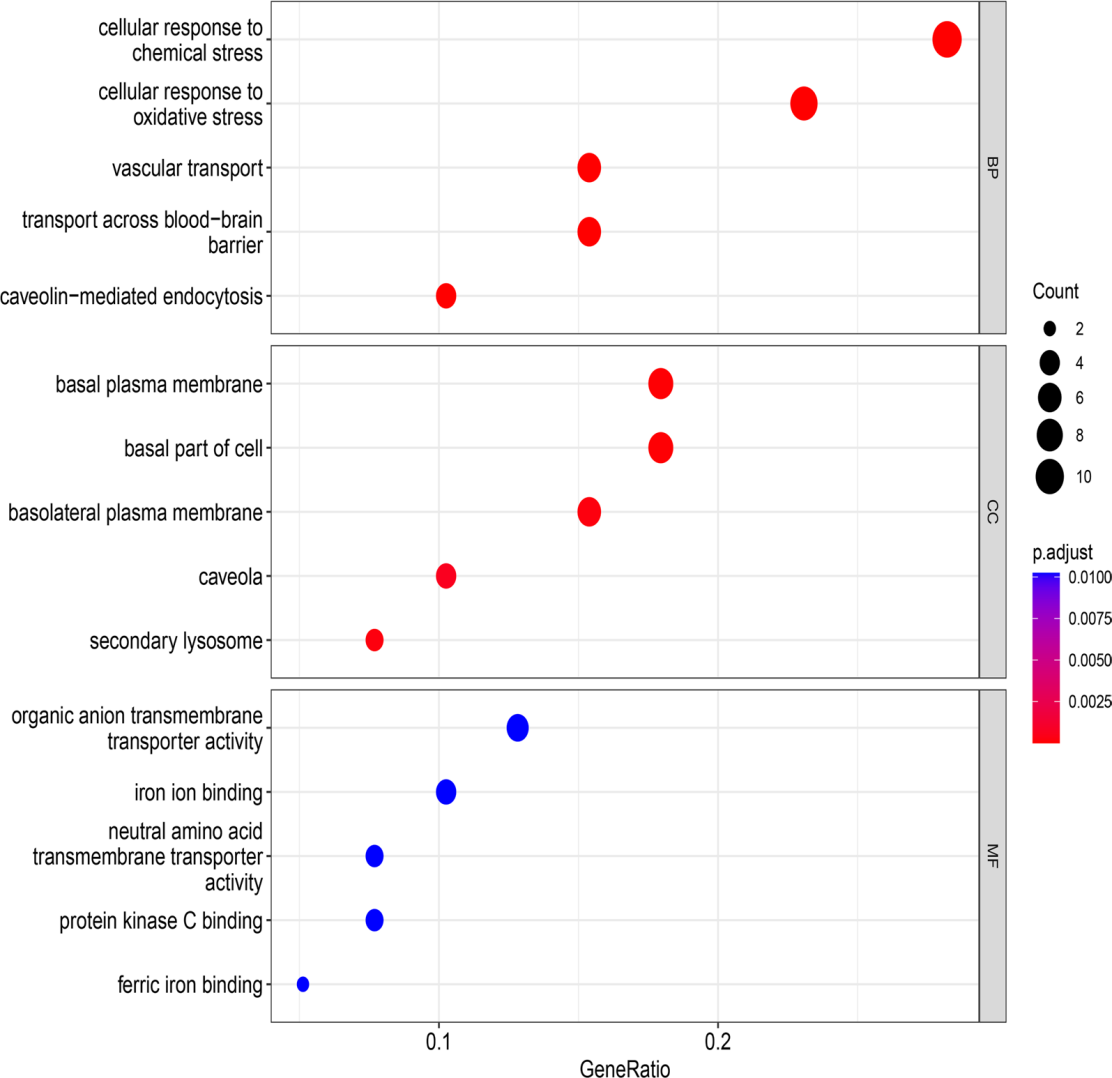
Results of cuproptosis-related ferroptosis genes from GO analysis (BP, CC, and MF represent biological processes, cellular component, and molecular function, respectively)

Based on our summary and considerations, we analyzed the expression level of cuproptosis-related ferroptosis genes in liver cancer datasets by using The Cancer Genome Atlas Program (TCGA, https://www.cancer.gov/ccg/research/genome-sequencing/tcga) (Fig. 2). In addition, we used bioinformatics to mine the cuproptosis-related ferroptosis genes and then performed Gene Ontology (GO) and Kyoto Encyclopedia of Genes and Genomes (KEGG) analyses to explore their potential function. In the KEGG analysis, cuproptosis-related ferroptosis genes were found in the “Central carbon metabolism in cancer” (p = 1.40E−10) and the “VEGF signaling pathway” (p = 2.53E−06) (Fig. 3). At the biological process (BP) level of GO analysis, the genes were mainly involved in the “cellular response to chemical stress” (p = 6.78E−11), “cellular response to oxidative stress” (p = 5.54E−09) and “vascular transport” (p = 2.30E−08). At the cellular component (CC) level of GO analysis, the genes were mainly involved in the “basal plasma membrane” (p = 5.57E−07), “basal part of cell” (p = 8.82E−07) and “basolateral plasma membrane” (p = 5.21E−06). At the molecular function (MF) level of GO analysis, the genes mainly involved in “organic anion transmembrane transporter activity” (p = 4.78E−0.5), “iron ion binding” (p = 0.000299) and “neutral amino acid transmembrane transporter activity” (p = 0.000109) (Fig. 4). These indicates that cuproptosis and ferroptosis may be involved in the regulation of mitochondrial function and tumor microenvironment together by Vascular function, ion binding, and plasma membrane.

Furthermore, Xue et al. found that copper promotes the formation of GPX4 aggregation, and then GPX4 is degraded by autophagy and promotes ferroptosis [42]. This provides the basis for cuproptosis combined with ferroptosis to antitumor and treat diseases.

In this review, the mechanism of action of cuproptosis and ferroptosis, how cuproptosis and ferroptosis inhibits the occurrence of liver cancer, and how cuproptosis and ferroptosis play a role in the immune microenvironment of liver cancer are summarized. Based on the above, we hypothesize the cuproptosis and ferroptosis are involved in the immunomodulation of liver cancer, but there is a lack of experimental verification. The relevant research mechanism of cuproptosis combined with ferroptosis in liver cancer is still relatively rare, and we hope that more in-depth research can be carried out in the future. For example, the combination of cuproptosis and ferroptosis affects immunity to liver cancer. Altogether, the research on cuproptosis combined with ferroptosis for liver cancer is still in the research growth period, and there is much room for its academic promotion and application in tumor therapy.

## Data Availability

The datasets generated during and/or analyzed during the current study are available from the corresponding author upon reasonable request.
